# Herbert W. Kendall

**Published:** 1907-03

**Authors:** 


### Obituary Notices.

### HERBERT W. KENDALL, F.R.C.S.

BY the death, on December 23rd last, of Mr. H. W. Kendall, of Redland, the profession has lost a colleague who, both as a student and practitioner, was characterised by steadfast integrity of character and aim. He passed away at the early age of 39, after an illness of three weeks' duration, the end coming suddenly from pulmonary thrombosis dependent on lung trouble, following an attack of appendicitis, and at a time when he was showing signs of apparent recovery; his sudden death was a termination somewhat unexpected by his friends, and by the several medical and surgical colleagues who anxiously attended him.

As a general practitioner, he spared no time or pains for rich and poor alike. It was his very nature to be thorough in his work and kind in his ways, denying himself at the call of duty, and gaining the esteem of both patients and colleagues.

For several years past he was Honorary Surgeon to the Bristol Royal Hospital for Sick Children and Women; he was an able operator, and was much respected by the staff, committee, and nurses. His death will be a great loss to that institution, which he served so faithfully and well. He was also Medical Officer to the Maternity Home, and to the Surgical Aid Society; and, though holding several public appointments, he performed the duties of all thoroughly and creditably.

Mr. Kendall started practice in Bristol in 1895, after holding the posts of House Surgeon to the Great Northern Hospital, and of House Surgeon and Obstetric Officer to the Middlesex Hospital, where he studied in London. He leaves numerous professional and lay friends to mourn his loss.

